# Hiding the Source Based on Limited Flooding for Sensor Networks

**DOI:** 10.3390/s151129129

**Published:** 2015-11-17

**Authors:** Juan Chen, Zhengkui Lin, Ying Hu, Bailing Wang

**Affiliations:** 1 Department of Information Science and Technology, Dalian Maritime University, Dalian 116026, China; E-Mails:juanchencs@gmail.com (J.C.); dalianjx@163.com (Z.L.); huying@dlmu.edu.cn (B.W.); 2 Department of Computer Science, Harbin Institute of Technology, Weihai 264209, China

**Keywords:** wireless sensors network, internet of things, source location, privacy protection, security

## Abstract

Wireless sensor networks are widely used to monitor valuable objects such as rare animals or armies. Once an object is detected, the source, *i.e.*, the sensor nearest to the object, generates and periodically sends a packet about the object to the base station. Since attackers can capture the object by localizing the source, many protocols have been proposed to protect source location. Instead of transmitting the packet to the base station directly, typical source location protection protocols first transmit packets randomly for a few hops to a phantom location, and then forward the packets to the base station. The problem with these protocols is that the generated phantom locations are usually not only near the true source but also close to each other. As a result, attackers can easily trace a route back to the source from the phantom locations. To address the above problem, we propose a new protocol for source location protection based on limited flooding, named SLP. Compared with existing protocols, SLP can generate phantom locations that are not only far away from the source, but also widely distributed. It improves source location security significantly with low communication cost. We further propose a protocol, namely SLP-E, to protect source location against more powerful attackers with wider fields of vision. The performance of our SLP and SLP-E are validated by both theoretical analysis and simulation results.

## 1. Introduction

Wireless sensor networks (WSNs) are widely deployed in the Internet of Things (IoT) to monitor valuable objects such as rare animals or armies in battlefields [1]. Once an object is detected, the sensor nearest to the object, *i.e.*, the source, would periodically send information about the object to the base station [2]. Generally, an attacker could trace the source and then capture the valuable object by localizing the source, therefore, many protocols for source location protection are proposed to protect the object by preventing attackers from localizing the source.

A typical source location protection model is the panda-hunter model [3,4]. In this model, sensors are deployed to monitor pandas. Once a panda is monitored, the source would periodically generate and transmit packets through the sensors one by one to the base station. In order to capture the panda, the hunter starts from the base station and tries to locate the source by tracing the packets back hop by hop. The essential idea of existing source location protection protocols includes two major steps. First, a packet is transmitted from the source randomly to other sensors for *h*-hops, before it arrives at a location, called the phantom location. Second, the packet is transmitted from the phantom location to the base station through flooding or the shortest path routing [5,6,7]. The first step should generate phantom locations that are not only widely distributed, but also far away from the source [8], so that it would be more difficult for an attacker to trace back to the source even if it has located the phantom location. However, the phantom locations generated by existing works [3,4,9] usually gather in two regions (we will prove this point in Section 2) and they are not guaranteed to be far away from the source. As a result, the random *h*-hops in the first step cannot effectively misguide an attacker. To address the above limitations, we first propose a protocol for source location protection based on limited flooding, named SLP, which improves source location security with low communication cost. Considering more powerful attackers with wider fields of vision, we further propose an enhanced protocol for source location protection, named SLP-E. In a nutshell, our contributions are mainly twofold: first, we propose a source location protection protocol named SLP to generate phantom locations that are not only far away from the source, but also widely distributed. Both theoretical analysis and simulation results show that SLP provides more secure source locations than typical source location protection protocols [4,9]. The average safety period is increased by nearly an order of magnitude.

Second, we propose an enhanced source location protection protocol based on SLP, named SLP-E, to protect against more powerful attackers with wider fields of vision. SLP-E further improves the source location security with low communication cost. The performance of our SLP-E is validated by theory and simulation, respectively.

The remainder of this paper is organized as follows: in Section 2, we review the previous work about source location protection in WSNs and investigate the limitations of previous works. Section 3 provides the problem definition. Section 4 and Section 5 introduce in detail SLP and SLP-E, respectively. The communication cost and security performance for our protocols are analyzed in Section 6. Simulation results are given in Section 7. Finally, Section 8 concludes this paper.

## 2. Related Work

Existing works on source location protection can be divided into two major categories: source location protection against the global attackers (SLP-GA) [10,11,12] and source location protection against local attackers (SLP-LA) [3,4,9,13].

To defend against global attackers, Mehta *et al.* [10] presented the FitProbRate scheme. By controlling the sending rate of packets, FitProbRate enhances source anonymity and meanwhile decreases packet transmission latency. Yang *et* *al.* [11] focused on the tradeoff between security and performance, and provide source anonymity under a global attack. The proposed model can significantly reduce the packet reporting latency based on its “*indistinguishable even*ability”. However, this model cannot capture the source information leakage. Cuellar *et al.* [12] provides a statistical framework that is stronger than the “*indistinguishable event*” ability for modeling, analyzing, and evaluating anonymity in sensor networks. All the above works have limitations. First, all sensors in the above work send lots of fakes packets, which incurs a considerable energy consumption and increases the probability of packet collision (and consequently the packet loss). Second, it is reasonably difficult for an attacker to capture the traffic over a large scale area in the real world. For example, to monitor the whole traffic over the Wolong Panda Reserve in China which covers about two million square kilometers is difficult.

To defend against local attackers, Ozturk *et* *al.* [3] propose a phantom routing protocol for source location protection. The phantom routing protocol includes two major steps. In the first step, each packet is transmitted from the source by random *h*-hops and then arrives at a phantom location. In the second step, each packet is transmitted from the phantom location to the base station through flooding or the shortest path routing. If phantom locations are far away from the source, attackers can be misled away from the source more effectively. However, theoretical analysis presented in [13] indicates that the probability for the distance between phantom location and source to be no more than *h/5* is *p =* 1 *−* *e^−h/25^*. Apparently, *p* approaches 1 if *h* is large enough. In order to generate phantom locations that are far away from the source, Kamat *et al.* [4] proposed a directed random routing protocol. In this protocol, each sensor is designated with a *D_bs_* that is equal to the shortest distance from the sensor to the base station. As sensors are assumed to be distributed evenly, the number of hops is adopted to measure the distance between two sensors. In the first step, given any sensor, say *u*, its neighbors are divided into two sets, the parent set and the child set, satisfying any sensor in the parent set has a smaller *D_bs_* and any sensor in the child set has a larger *D_bs_* than that of *u*. Once the source generates a packet, it first determines a set (*i.e.*, the parent set or the child set) and then forwards the packet to a random sensor of the set. When a sensor receives the packet, it continues forwarding the packet to another sensor of the child set (which is determined by the source). This packet-forwarding process will be repeated until the packet has been forwarded *h-*hops. In this way, each packet is guaranteed to be transmitted far from or near the base station within the *h*-hops. However, the phantom locations generated by [3,4,13] tend to gather in two regions (to be proved by Lemma 1). As a result, the attacker is likely to be led to the source as he can reach to one of the two regions easily.

Differing from above work, Wang *et al.* [9] considered a more powerful attacker who can observe all the sensors within *r* (*r* ≥ 0) hops rather than only one hop away from him. For this attacker, the source is revealed once he traces the signals back to the unsafe area within *r* hops from the source. Theoretical analysis in [9] further indicates that if packets are routed through the unsafe area, the attacker can trace back to the source quickly. Wang *et al.* [9] proposed a protocol based on angle area to protect the source, which has several limitations: (1) determining the angle area incurs extra computational cost; (2) the generated phantom locations still gather with a high probability in two regions because each next forwarding sensor is chosen based on angle area; and (3) packets are not guaranteed to be routed bypassing the unsafe area. In [14] an opportunistic routing protocol (OpRo) is used to enhance source-location privacy. This work introduces three schemes, *i.e.*, non-repeating opportunistic routing, opportunistic routing with random delay, and opportunistic routing with random relay to protect the source. Based on this work, Spachos *et al.* [15] further proposed an Angle-based Dynamic Routing Scheme (ADRS) to enhance the source location privacy. ADRS employs the location information of nodes and calculates an inclination angle to avoid cycles around the source, but it cannot be applied to the network without knowing each node’s location.

Another solution against traffic analysis is introduced in Reed *et* *al.* [16]. In his work, Reed *et al.* [16] made use of the principle of anonymous connections for the Internet, in which the traffic between sender and receiver is obfuscated and routed through several different stations. This principle was realized with the concept of onion routing. Later on, Reiter *et al.* [17] provide a solution called CROWDS, which uses anonymity sets, which include different degrees of anonymity, directly. Solutions in this category hide either the identity or the location of a sensor node. Other typical work in this category includes the anonymous communication scheme [18], the anonymous path routing [19], and the hashing based ID randomization [20].

Note that anonymity and unobservability may not be enough to protect a source’s location. In fact, the source can be also endangered if an adversary is able to find the previous locations of a node, which allows the adversary to create a trajectory and forecast the next position [21]. To address the above limitations, we propose our new protocols SLP and SLP-E, to enhance the source location protection with low communication cost.

## 3. Problem Definition

### 3.1. Network Model

We define our problem based on the classical panda-hunter game network model [4,9]. In the panda-hunter game model, a sensor network is used to monitor the pandas’ activity. Once a panda is detected, the nearest sensor would become the source. The source would then generate encrypted event packets about the panda and send them to the sink periodically. Attackers such as hunters try to localize the source, so as to capture the panda by tracing back packets hop-by-hop from the sink. The purpose of our protocol is to protect the panda by concealing the source location. Specifically, we make the following assumptions about our network model:

- Sensors are deployed evenly over the whole network. Any two sensors can communicate with each other hop-by-hop.
- Only one sink exists as the controller of the network. The sink collects or retrieves data from sensors from time to time.

### 3.2. Attack Model

The attacker is usually equipped with high-end hardware in the real world, due to the high profitability of panda hunting, therefore, we define the characteristics of the attacker as follows.

*Higher-functioning Hardware*: The attacker has powerful memory capacity and computation ability. He can observe the packet sender by wireless radio frequency techniques and move to the packet sender immediately.

*Passive Traffic Monitoring*. The attacker is equipped with supporting devices, such as antenna and spectrum analyzers, so that he can measure the arrival angle of a packet as well as the strength of the signal. From these two measurements, after he overhears a signal, he is able to estimate the location of the sending node. We assume the hearing radius of the attacker is equal to the sensor’s transmission range [4,9]. He can capture packets but cannot decrypt and understand the packets. Because a packet is transmitted as a local broadcast, an attacker overhearing the transmission can only tell the location of the immediate transmitter but not the location of the node that is receiving the packet. Let us illustrate how an attacker traces packets in a sensor network by an example. Suppose the attacker resides at node A. He can therefore monitor the packet transmissions from nodes within A’s transmission range including A, B, C and D. He overhears a transmission made from node B. Shortly after, he overhears a transmission from node A. Based on the above sequence of transmissions, the attacker learns that a packet was sent from B to A and then to C. The attacker will move to B, hoping that he is one hop closer to the source. The movement of the attacker is far slower than the movement of a packet in the network. There are two types of attackers according to the applied tracing strategies [4], the patient attacker and the cautious attacker. The former keeps waiting until it observes a packet being sent and then moves to the packet sender. The latter remembers its own path. It stays at each position for at most a maximum time period, and will return to the previous location (the last hop) once it has stayed for the fixed time period without observing anything. Since experimental results [4] demonstrate that the patient attacker has a stronger attacking ability than the cautious attacker, we only study the patient attacker in this paper.

### 3.3. Security Assumption

The base station generates a public/private key pair and distributes the public key to all the sensors. We also assume that the base station is safe and cannot be captured by the attacker. For example, it is reasonable to assume that the hunter does want to not capture the manager of the zoo.

## 4. Protocol for Source Location Protection Based on Limited Flooding

The base station initializes the network by broadcast in a similar way as described by Kang in [22]. After the network initialization, every sensor knows its neighbors and its shortest distance away from the base station (denoted by *D_bs_*). When the source (e.g., *s*) detects the panda, *s* starts a flooding within a limited area *Q*, which covers the area within *h*-hops from *s*. The flooding packet will be transmitted to every sensor in *Q*. After the flooding, each sensor in *Q* acquires its shortest distance from the *s* (denoted by *D_s_*). After that, the source generates and sends a packet to the base station by a *h*-directed routing and then the shortest path routing. In particular, in the *h*-directed routing, a packet will always be sent to a neighbor whose *D_s_* is larger than that of the current sensor (*i.e.*, the sensor that forwards this packet to the neighbor). After *h* directed transmissions, the packet reaches a location called the phantom location. For the *h*-directed routing, the phantom locations obtained for the packets are expected to be widely distributed and also far from the source. Finally, the packets will be transmitted from the phantom locations to the base station through the shortest path routing. During this process, the packet will always be forwarded to a neighbor which is nearer to the base station than the current sensor. Our proposed SLP includes four phases: network initialization, *h*-hops limited flooding initialized from the source, *h*-directed routing, and the shortest path routing. Table 1 lists the notations used in this paper.

**Table 1:** Notations used in this paper.

(K_pub_, K_pri_)	The public/private key pair for packet encryption and decryption
E_Kpub_(m)	Encrypt packet m by public key K_pub_
bs	Base station
Hop_u,v_	The shortest distance from sensor u to sensor v measured by hops
p_i_	Phantom location
u.neighbor	The set of sensor u’ neighbors
u.set_parent	{v|v∈u.neighbor∩Hop_v,b_ < Hop_u,b_}
r	The visual radius for the attacker
h	The random directed hops
$Hop_{uv}$	The hops from sensor u to sensor v along the inferior arc uv
H	The shortest distance from source to the base station measured by hops
R	Transmission range of a sensor
$SP_{u,v}$	A path from sensor u to v

### 4.1. Network Initialization

As the foundation of the source location protection protocol, network initialization is responsible for distributing the keys, discovering neighbors, and helping each sensor to acquire its *D_bs_*. After the network initialization, the base station stores a public/private key pair (*K_pub_*, *K_pri_*), and each sensor obtains the public key *K_pub_* distributed from the base station. To defend against the active attack, each packet is encrypted by the public key before being transmitted by the source. After the sensors are deployed, base station broadcasts a beacon packet *BM* = {*BRO_BASE*, *ID*, *hop_b*}, where *BRO_BASE* denotes the packet type, *ID* field is the unique identifier of the packet sender, *hop_bs* records the transmission times of the packet and is initialized as 0. For any sensor *u*, if *u* receives *BM* for the first time, it first increases the *hop_bs* by 1 and updates *Hop_u,bs_* as equal to *hop_bs*. After that, *u* will broadcast this packet.

### 4.2. h-Hops Limited Flooding Initialized from the Source

The limited flooding is started by the source *s* within the area *h-*hops away from *s*. This has two purposes: (1) initialize the nodes within the flooding area about their distance to the source; and (2) exchange the distance information of each node with their neighbors.

Once a sensor detects the panda, it becomes the source. The source starts a flooding by sending a flooding packet {*BRO_SOURCE*, *ID*, *hop_s*} to all its neighbors, where *BRO_SOURCE* and *ID* denote the packet type and the unique identifier of the packet sender, respectively. The *hop_s* is initialized to 0 and increased by 1 after each transmission. The flooding packet will be discarded once *hop_s* achieves *h*. Different from the broadcast performed in the network initialization process, here, *hop_s* is used to record the shortest distance from the current sensor to the source instead of the base station. Since the flooding packet will only be transmitted when *hop_s* ≤ *h*, the flooding area is limited within *h*-hops from the source. After the flooding, each sensor in the flooding area has its *D_s_* and knows the *D_s_* of each of its neighbors. After that, each sensor within the flooding area, say *u*, can construct a set *Đ*, which includes all its neighbors each of which has a larger *D_s_* than that of *u*.

### 4.3. h-Directed Routing

After the *h*-hops limited flooding process, the packet will be transmitted for *h*-hops, where each hop goes farther away from the source. The *h*-directed routing aims at generating phantom locations that are widely distributed and also as far as possible from the source. To this end, each packet should be transmitted far away from the source for each hop. SLP always sends the packet to a random sensor of *Đ*. Therefore, the phantom locations generated are scattered and far away from the source. 

Specifically, the source generates a packet to be transmitted for *h-*hops during this phase as {*EVENT*, *E_Kpub_*(*m*), *hop_rand*, *Next_hop_id*}, where *EVENT* is the packet type and *E_Kpub_*(*m*) denotes the event data *m*, which has been encrypted by the public key *K_pub_*. *hop_rand* is used to record the packet transmission times and initialized as 0. It will be increased by 1 after each transmission. The *h*-directed routing phase will terminate as soon as *hop_rand* reaches *h*, *Next_hop_id* is the unique identifier of the next forwarding sensor. Once a sensor (*i.e.*, the current sensor) receives a packet and finds the packet satisfies: (1) the *Next_hop_id* equals to its ID; (2) the packet type is *EVENT*; and (3) *hop_rand* < *h*, the sensor will choose a sensor randomly from *Đ* and forward the packet to the sensor. The packet forwarding process repeats until *hop_rand* has increased to *h*. If the current sensor *u* selects a neighbor *v* as its next forwarding sensor for a packet and the two sensors satisfies *Hop_v,s_* *−* *Hop_u,s_* = 1, the packet is believed to have been sent far away from the source *s*. Thus, if the next forwarding sensor is selected from *Đ* of *u*, the packet is assured to be sent far away from the source. In addition, since the next forwarding sensor is chosen randomly from *Đ*, the phantom locations obtained from SLP are widely and evenly distributed in the probabilistic sense. SLP ensures it is difficult for an attacker to trace to the source even if he has reached to a phantom location. 

### 4.4. The Shortest Path Routing

After the *h*-directed routing, each packet will be routed following the shortest path from the phantom location to the base station. Specifically, the current sensor will always send the packet to a neighbor who has a smaller *D_bs_* than the *D_bs_* of the current sensor. The packet transmission process will be repeated until the packet reaches the base station.

## 5. Protocol for Source Location Protection Enhancement Based on Limited Flooding

To defend against attackers with more powerful monitoring ability than that assumed in defining SLP, we further propose an enhanced protocol for source location protection (SLP-E). We assume that the attacker can observe the sensors within *r* (0 < *r* < *h*) hops rather than only one hop away from it. As a result, once the attacker traces to the unsafe area, which is within r hops from the source, the source is revealed.

**Definition 1.**  The inefficient path is the path going through the unsafe area.

**Figure 1:**
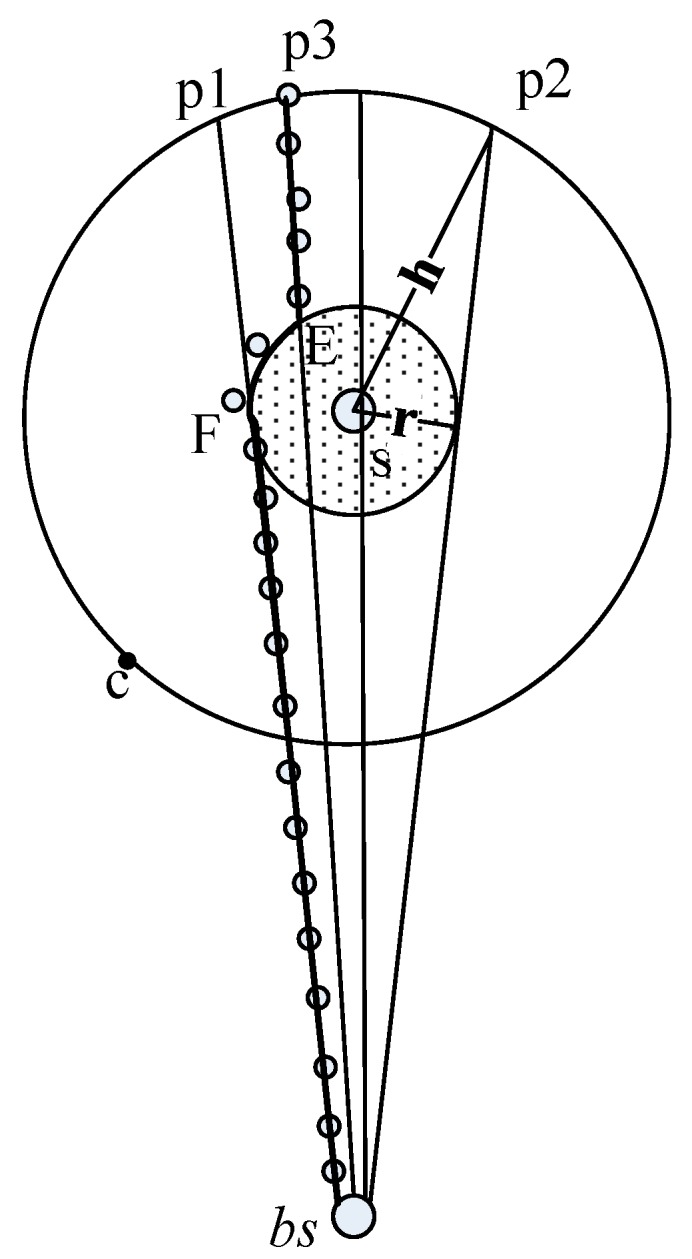
The shortest path bypassing the unsafe area.

As can be seen in Figure 1, during the shortest path routing process (see Section 4), if the phantom location lies on $p_{1}p_{3}p_{2}$, the path will go through the unsafe area and thus an inefficient path is generated. Wang *et al.* [9] proves that for a large sensor network of evenly distributed sensors, the probability of inefficient path generation is:

(1) (arcsin(r/H)+arcsin(r/h))/π

Since the attacker can trace the source quickly through an inefficient path, it is important to avoid generating inefficient paths for source location security enhancement. Based on the above discussion, we propose SLP-E to enhance the source location security by avoiding inefficient path generation. SLP-E is based on SLP, but differs from SLP in the following aspects. First, during the network initialization phase, each sensor is preloaded with a new parameter *r*, where *r* stands for the field of vision of the attacker. Second, during the *h*-hops limited flooding phase, the sensors within the unsafe area are marked. Finally, during the shortest path routing phase, a packet is routed to the base station bypassing the sensors in the unsafe area. SLP-E can void generating inefficient path and thus improve the source location security.

During the limited flooding phase of SLP-E, sensors within the unsafe area are marked, where each sensor is preloaded with a parameter *v_isual_* (initialized to 0). After the limited flooding, *v_isual_* will be updated to 1 if the sensor is within the unsafe area.

After the limited flooding, the source will route the packet to the phantom location by the *h*-directed routing introduced in Section 4. Figure 1 shows the packet will be transmitted *h*-hops away from the source before arriving at a phantom location such as *p*_1_.

During the shortest path routing process, the packet will be routed from the phantom location to the base station bypassing the unsafe area. As a result, the packet will always be transmitted to a sensor with *v_isual_* = 0. Specifically, the current sensor, say *u*, first classifies its neighbors into two sets: V_1_ and V_0_, where V_1_ includes the sensors with *v_isual_* = 1 and V_0_ the sensors with *v_isual_* = 0. Then, *u* will forward the packet to a neighbor that belongs to V_0_ ∩ *u.set_parent*. Since all the sensors of V_0_ are outside the unsafe area, the packets will be routed to the base station without passing through the unsafe area. Figure 1 shows that once the packet arrives at the phantom location (e.g., *p*_3_), it will take three sub-paths, namely $SP_{p_{3},E}$, $SP_{\overset{⌢}{EF}}$, and $SP_{F,bs}$, to arrive the base station, where $SP_{p_{3},E}$ denotes the shortest path between *p*_3_ and *E*. *E* is the intersection of circle G and the line passing through *p*_3_ and *bs*, where *G* is a circle with the center point of *s* and the radius of *r*. Similarly, $SP_{F,bs}$ denotes the shortest path between *F* and *bs*, and $SP_{\overset{⌢}{EF}}$ denotes the path from *E* to *F* along the inferior arc EF.

## 6. Performance Analysis

In this section we evaluate the performance of SLP and SLP-E in terms of communication cost, computational cost, security analysis, and communication cost *vs*. security.

### 6.1. Communication Cost 

The communication cost is measured by the total number of packet transmissions [4,9]. The communication cost of both SLP and SLP-E is the result of four phases, *i.e.*, the broadcast by the base station during the network initialization, the *h*-hops limited flooding initialized from the source, the *h*-directed routing, and the shortest path routing. As the communication cost of SLP and SLP-E resulting from the base station broadcast is the same as that of the existing work [3,4,9,13], we focus on studying the communication cost resulting from the last three phases. In addition, since *h* ≪ $\sqrt{S/\pi }$, where *S* denotes the area of the network, the communication cost for the limited flooding phase is extremely low. Specifically, the communication overhead of limited hop flooding is $C_{L}=\pi h^{2}\rho$, where ρ denotes the node density. We also have the n=Sρ. Then, we obtain $C_{L}=(\pi n/S)h^{2}$. Since *h* ≪ $\sqrt{S/\pi }$, then we have $C_{L}=(\pi n/S)h^{2}\ll$ = *n*. Since the communication cost of limited flooding is extremely low. Therefore, we don’t consider the communication cost resulted from this phase as well.

According to the above analysis, we will only discuss the communication cost from the *h*-directed routing and the shortest path routing phases. Below, we will discuss the communication cost of SLP and SLP-E, respectively.

(1) Communication Cost for SLP

**Figure 2:**
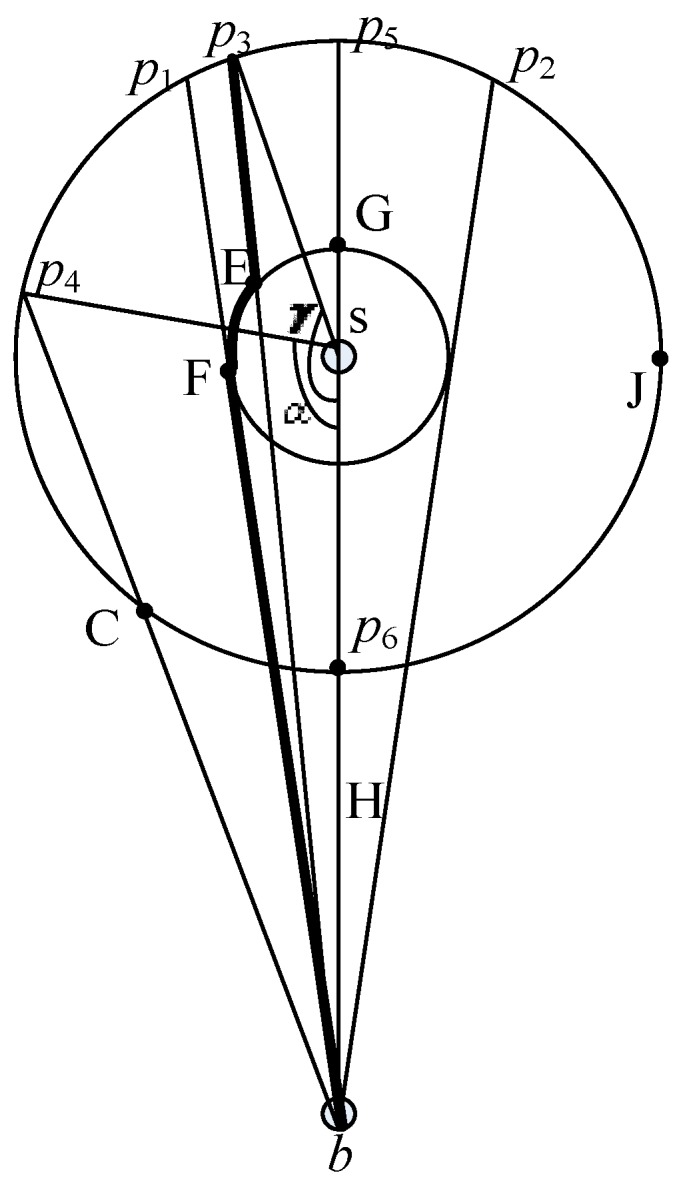
The path from the phantom location to the base station.

Figure 2 shows the idea of SLP using a circle *I* with a center point *s* and a radius *h*. In SLP, a packet is first transmitted to a phantom location (e.g., *p*_4_ ∈ *I*) after *h*-directed routing. Then, the packet is transmitted from *p*_4_ to the base station by the shortest path routing. Hence, the communication cost of SLP is *Hop_s,p_*_4_ *+ Hop_p_*_4*,b*_, where *Hop_s,p4_* *= h*.

According to the cosine theorem, we also have *Hop_p_*_4_*_,b_* *=* $\sqrt{h^{2}+H^{2}-2hH\cos\alpha }$ for the triangle with *s*, *p_4_* and *b*. Therefore, we obtain the communication of SLP as follows:

(2) $$h+\sqrt{h^{2}+H^{2}-2hH\cos\alpha }$$

where *α* ϵ (0, *2π*]. According to Equation (2), the communication cost of SLP achieves the maximum value of *H* + 2*h* when *α* = π. Figure 2 shows that in this case, the packet first reaches *p*_5_ through the *h*-directed routing, and then the base station through the shortest path routing. Similarly, the communication cost for SLP achieves the minimum value of *H* when *α = 2π*. Under this situation, the packet first reaches *p*_6_ through the *h*-directed routing, and then the base station through the shortest path routing. To form a more general case, we assume that *p*_3_ is a random point on circle *I* (as shown in Figure 2). We calculate the average hops from *p*_3_ to the base station for the more general case as:

(3) $$\int_{0}^{\pi }\sqrt{h^{2}+H^{2}-2hH\cos\alpha }/\pi d\alpha$$

The average communication cost of SLP is thus obtained by:

(4) $$h+\int_{0}^{\pi }\sqrt{h^{2}+H^{2}-2hH\cos\alpha }/\pi d\alpha$$

(2) Communication Cost for SLP-E

The communication cost of SLP-E is calculated for the following two situations:

(a) If the phantom location, namely $p_{4}$, is on arc $p_{1}Cp_{2}$, the communication cost of SLP-E is the same as that of SLP:

$${Hop}_{s,p4}+{Hop}_{p4,b}=h\;+\sqrt{h^{2}+H^{2}-2hH\cos\alpha }$$.

(b) If the phantom location, namely $p_{3}$, is on arc $p_{1}p_{5}p_{2}$, the packet will be first routed to E and then to F along the arc EF. Finally, the packet will arrive at the base station through the shortest path from F to b. Therefore, the communication cost of SLP-E is Hop_s,p3_ + Hop_p3,E_ + $Hop_{EF}$+ Hop_F,b_, where Hop_s,p3_ = *h* and Hop_F,b_ = $\sqrt{H^{2}-r^{2}}$. For $\Delta p_{3}sb$, we have Hop_p3,b_ = $\sqrt{h^{2}+H^{2}-2hH\cos\gamma }$. Given $∠1=∠bp_{3}s$, $∠2=∠sbp_{3}$, ∠3=∠sEb and $∠4=∠Esp_{3}$, we have $∠1=\arcsin(H\sin\gamma /Hop_{p_{3},b})$ and $∠2=\arcsin(h\sin\gamma /Hop_{p_{3},b})$ for $\Delta p_{3}sb$, according to the sine theorem. Similarly, we have ∠3=arcsin(Hsin∠2/r) for ΔEsb and ∠4=∠3−∠1 for $\Delta p_{3}Es$. We finally obtain Hop_p3,E_ = $\sqrt{h^{2}+r^{2}-2hr\cos∠4}$ according to the cosine theorem and further obtain $Hop_{EF}$ = (γ−arccos(r/H)−∠4)r. According to (a) and (b), given that p_4_ is a point on arc $p_{1}Cp_{2}$ and p_3_ is a point on the inferior arc $p_{1}p_{5}p_{2}$, we have α ∈ [0, arcos(r/H) + arcos(r/h)) and γ ∈ [arcos(r/H) + arcos(r/h), π]. Below we will discuss the maximum, minimum and average communication cost of SLP-E.

- When *γ=π*, SLP-E achieves the maximum communication cost of $2h+(\pi -1-\arccos(r/H))r+\sqrt{H^{2}-r^{2}}$. In this case, the packet first arrives at the phantom location *p*_5_ and then at the base station through the line passing through *p*_5_ and *b*.
- When *α* = 0, SLP-E achieves the minimum communication cost of *H*. In this case, the packet first arrives at the phantom location *p*_6_ and then at the base station through the line passing through *p*_6_ and *b*.
- The average communication cost of SLP-E is:
  $$h+\sqrt{H^{2}-r^{2}}(\arcsin(r/H)+\arcsin(r/h))/\pi +\int_{0}^{\arccos(r/H)+\arccos(r/h)}(\sqrt{h^{2}+H^{2}-2hH\cos\alpha })/\pi d\alpha +\int_{\arccos(r/H)+\arccos(r/h)}^{\pi }(\sqrt{h^{2}+r^{2}-2hr\cos∠4}+(\alpha -\arccos(r/H)-∠4)r)/\pi d\alpha .$$

(3) Commucniaton Cost Comparison between SLP and SLP-E

The communication cost of SLP and SLP-E are the same if the phantom location lies on $p_{1}Cp_{2}$, according to the previous discussion. However, if the phantom location lies on $p_{1}p_{5}p_{2}$, the communication cost of SLP-E becomes higher than that of SLP. Specifically, given the increased amount of communication cost *f*, we have

(a) *f* *= 0 if p*_3_ *is on* $p_{1}Cp_{2}$*;*
(b) *f* = $Hop_{EF}$+ *Hop_F,b_*-*Hop_E,b_* if *p*_3_ is on $p_{1}p_{5}p_{2}$.

Let $\beta_{1}=∠Esb$ and $\beta_{2}=∠Fsb=\arccos(r/H)$, we have $Hop_{\overset{⌢}{EF}}=(\beta_{1}-\beta_{2})r$ and *Hop_E,b_* = $\sqrt{H^{2}+r^{2}-2Hr\cos\beta_{1}}$, according to the cosine theorem for ΔEsb. If *p*_3_ lies on $p_{1}p_{5}p_{2}$, we can obtain *f* = $(\beta_{1}-\beta_{2})r$ + $\sqrt{H^{2}-r^{2}}$ − $\sqrt{H^{2}+r^{2}-2Hr\cos\beta_{1}}$ and the first derivation of *f*,

$$f^{\prime }=\partial f/\partial \beta_{1}=(r/\sqrt{H^{2}+r^{2}-2Hr\cos\beta_{1}})(\sqrt{H^{2}+r^{2}-2Hr\cos\beta_{1}}-H\sin\beta_{1}).$$

Apparently, *f*’ ≥ 0 because *β*_1_ ∈ [*β*_2_, *π*]. In the worst case, *f*’ achieves the maximum value *f_max_* = $(\pi -1-\beta_{2})r$ + $\sqrt{H^{2}-r^{2}}$ *− H* if and only if *p*_3_ lies on *p*_5_.

As shown in Figure 2, the communication cost for SLP-E increases that of SLP averagely by:

(5) $$f_{avg}=(\int_{\arccos(r/H)+\arccos(r/h)}^{\pi }f_{\max}d\alpha +\int_{0}^{\arccos(r/H)+\arccos(r/h)}0d\alpha )/\pi =(\;f_{\max}/\pi )[\pi -\arccos(r/H)-\arccos(r/h)]$$

where *r/h* ∈ (0, 1) and *r/H* ∈ (0, 1]. *f_avg_* grows as *h* decreases according to Equation (4). *f_avg_* achieves the maximum value when *h = r*. Figure 2 shows how *f_avg_* varied with different *r/H* when *h = r* and *H* = 100.

Since the communication radius for existing sensors such as a Mica [23] is almost 60 m, the visible area of the attacker is extremely large if *r* is set to 6. Therefore, we let *r* ≤ 6 in this paper. For a large sensor networks, *r/H* is usually no more than 1/5 [9] and therefore we have *f_avg_* = 8. We can see from Table 2 that compared with SLP, the increased amount of communication cost for SLP-E is acceptable.

**Table 2:** The increased amount of communication cost on average.

***r/H***	1/2	1/5	1/10	1/15	1/20
***f_avg_***	28	8	3	2	1

### 6.2. Computation Cost

Compared with typical source location protection protocols (the phantom single-path and PRLA [4,9]) and the protocols described in [24] (also called general protocols), our protocols additionally provide security protection for the source. Considering the attacker with ordinary field of vision (*i.e.*, the attacker can only observe the sensors within only one hop’s distance), both the proposed protocols and the typical source location protection protocols incur no extra computational cost when compared with general protocols [24]. Considering the situation with more powerful attackers as described in Section 5, to protect the source, each sensor within *h*-hops from the source needs to calculate a value fort the next forwarding sensor selection in PRLA. Given the sensor density *ρ* and the communication radius of sensor *R*, there are Л*h^2^ R^2^ρ* extra calculations for PRLA. However, both the proposed protocols and the phantom single-path incur no extra computation cost when compared with other existing protocols.

### 6.3. Security Performance

In this section, we will analyze the security performance of the existing typical source location protection protocols [3,4,13] firstly and then our proposed protocols respectively.

(1) The Existing Typical Source Location Protection Protocols

The security performance of the protocols described in [3,4,13] improves as the generated phantom locations are more widely distributed. However, Lemma 1 proves that the phantom locations generated by [3,4,13] gather in two regions. As a result, the attacker is likely to be led to the source as he can reach to one of the two regions easily.

**Lemma 1.**  Suppose Hop_u,bs_ and Hop_v,bs_ are the shortest distance from sensor u and v to the base station respectively. The absolute difference between Hop_u,bs_ and Hop_v,bs_ is no more than 1, where v is u’s neighbor.

**Proof of Lemma 1.**  According to the broadcast scheme initialized by the base station in [3,4,9], for each *v* ∈ *u.neighbor*, we have that *Hop_v,u_ =* 1, where *u.neighbor* denotes the set of all neighbors of *u*. Apparently *Hop_v,bs_* − *Hop_u,bs_* ≤ 1 if *Hop_u,bs_* = *Hop_v,bs_*. If *Hop_u,bs_* > *Hop_v,bs_*, there is a path from *u* to base station through *v*, namely *R_u,bs_*. *R_u,bs_* consists of two parts: the shortest path from *u* to *v* and the shortest path from *v* to base station. Thus, we have *|R_u,bs_| =* 1 *+ Hop_v,bs_* and *|R_u,bs_*| ≥ *Hop_u,bs_*, where *|R_u,bs_*| is the length of path *R_u,bs_* measured by the number of hops. Finally, we have 1 *+ Hop_v,bs_* ≥ *Hop_u,b_* and *Hop_u,bs_ − Hop_v,bs_* ≤ 1. Similarly, we can prove that *Hop_v,b_ − Hop_u,bs_* ≤ 1 if *Hop_u,bs_* < *Hop_v,bs_*. In summary, we have |*Hop_v,bs_* − *Hop_u,bs_*| ≤ 1.

**Theorem 1.**  If the packet is forwarded to a sensor randomly chosen from the set determined by the source (i.e., parent set or child set of the current sensor), all phantom locations will gather in two regions after h-hops of transmissions.

**Figure 3:**
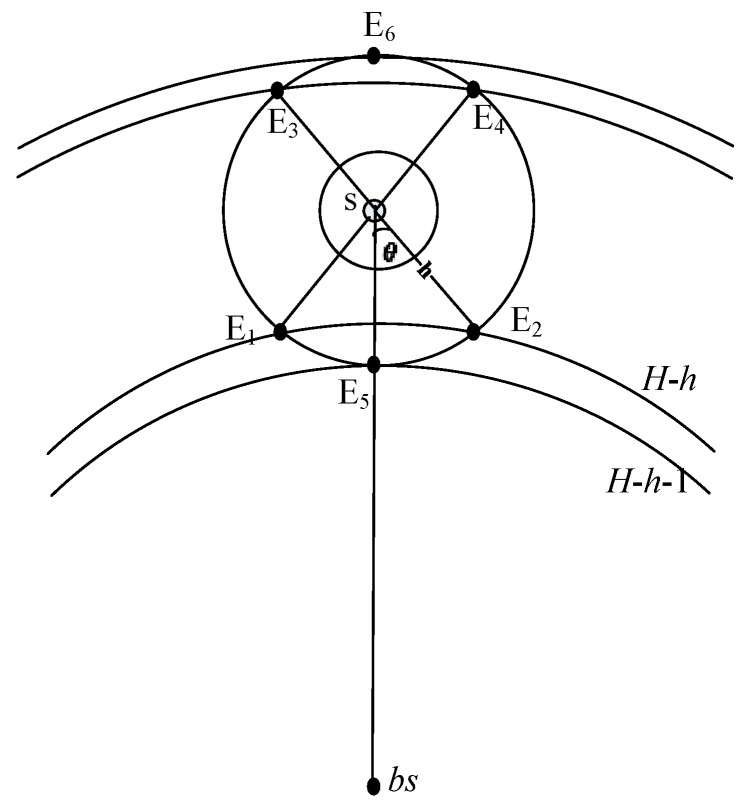
The phantom locations distribution.

**Proof of Theorem 1.**  Once a source determines the parent set as its forwarding set, the source *s* will send its packet to a sensor randomly chosen from its parent set. Similarly, the sensor responsible for the packet transmission will also forward the packet to a sensor randomly from its parent set. After *h* transmissions, the packet will reach the phantom location *p*, where the shortest distance between *p* and *s* measured by hops *Hop_p,s_* ≤ *h*. As is shown in Figure 3, *p* lies in the region $E_{1}E_{5}E_{2}$. According to Lemma 1, we have *Hop_p,bs_ = H* − *h*, where *H* denotes the shortest distance from the source to base station measured by the number of hops. Given that the center point of the base station and the radius of the *i-*th circle i*R (*I* = 1,2,3…), a sensor, say *u*, lies in the area between the *k*-th and the (*k* − 1)-th circle if *Hop_u,bs_* = *k* (*k =* 2,3…), where *R* denotes the transmission range of a sensor. Since any sensor within the transmission radius of the base station is one hop away from base station, given any phantom location, say *p*, it is located in the area between the (*H* − *h*)-th and (*H* − *h* − 1)-th circle. Similarly, we can prove that *p* lies in the region $E_{3}E_{6}E_{4}$ if *s* determines its child set as its forwarding set. In conclusion, we have that all phantom locations lie in the two regions including $E_{1}E_{5}E_{2}$ and $E_{3}E_{6}E_{4}$ with 4*θ* range, where *θ = arccos*((*h − 1*)*/h*) as shown in Figure 3.

(2) SLP and SLP-E against one Attacker

Different from existing work, SLP and SLP-E can enhance the source location protection by using widely distributed phantom locations, so even if the attacker can obtain our privacy protocols, it is difficult for him to localize the source by tracing multiple phantom locations. This is because the phantom locations are scattered in SLP and SLP-E. Thus, it takes a long time for the attacker to trace multiple phantom locations and hence the source. In a real situation, once the source detects an event, it will send several messages to the base station over a period of time, so the attacker can only trace messages hop by hop during that period of time. Since the phantom locations are scattered, SLP and SLP-E can extend the time that the attacker takes to locate the phantom locations and hence improve the source location’s security.

According to the *h*-directed routing phase introduced in Section 4, the more random the directed paths are, the wider distribution the phantom locations have and consequently the more difficult it is for the attacker to trace the source. Therefore, we measure the security performance of SLP by the number of random directed paths.

**Definition 2.**  The random directed path is defined as the path from the source to the random location by h-directed routing. Specifically, the number of random directed paths is used to measure the degree of geographical dispersion of phantom locations. This is because the phantom locations are more widely scattered as the number of random directed paths grows. As shown in Figure 3, any phantom location will be located at the circumference of the circle with center s and radius h. As a result, compared to previous work [4,9], the number of random directed paths for both SLP and SLP-E increases by Ф = 1 − 4θ/2Л = 1 − 2arccos((h − 1)/h). Furthermore, Table 3 shows the changes of Ф under varying h, where h ≥ 2. When h = 2, Ф increases by at least 33.33% compared to phantom single-path. Table 3 also shows that Ф increases as h increases. In particular, Ф increases to 88.36% when h = 60. We further obtain the mean value of Ф when h grows from 2 to 60, i.e., $1-\frac{2}{\pi }\frac{\sum_{h=2}^{60}\arccos(1-\frac{1}{h})}{60}$ = 79.83%. In conclusion, both SLP and SLP-E provide a stronger protection for source location because they increase the number of random directed paths significantly when compared with previous work [4,9].

**Table 3:** The percentage of random directed paths increase.

	h = 2	h = 20	h = 30	h = 40	h = 50	h = 60
**SLP**	33.33%	79.78%	83.52%	85.73%	87.15%	88.36%

Theorem 2 indicates that for an attacker with a wider field of vision, SLP-E can avoid the inefficient paths completely and hence further enhance the source location protection.

**Theorem 2.**  By marking sensors in the unsafe area and bypassing these marked sensors during the shortest path routing process, the inefficient path can be avoided completely for SLP-E.

**Proof of Theorem 2.**  In SLP-E, a sensor that transmits the packet for the first time during the shortest path routing process, say *u*, is out of the unsafe area because *h > r*. Node *u* forwards the packet to a neighbor that belongs to V_0_ ∩ *u.set_parent*. The packet forwarding process will be repeated until the packet reaches the base station. Since the packet is only transmitted by the sensors outside the unsafe area, no inefficient paths will be generated.

(3) SLP and SLP-E against Several Attackers

Assume that there are several attackers and only one source. Since it is difficult for attackers to trace the source from random locations during a large scale area, we assume that each attacker starts from the sink and tries to trace the sources one after another. Specifically, once a packet forwarding process is monitored within the transmission range of the sink, an attacker starts his tracing. SLP and SLP-E try to extend the time that each attacker takes to locate the phantom locations, so each attacker takes a long time to arrive at different scattered phantom locations. Since the transmission range of each attacker is the same as the sensor, extra time is needed for them to move together for information sharing. In a real situation, once the source detects an event, it will send several messages to the sink over a period of time, so attackers have to trace the source during that period of time. It is difficult for several attackers to work together to locate the source in SLP and SLP-E.

There are works focusing on dealing with a large number of collaborative attackers in the global traffic analysis attack. However, it is not reasonable to deploy lots of attackers to monitor all the packet transmissions over a large scale area in the real world. For example, as mentioned before, the Wolong Panda Reserve in China covers about two million square kilometers. For now, we have provided qualitative analysis results in this work, but leave more sophisticated analysis of the issue to our future work.

### 6.4. Communication Cost vs. Security 

The communication cost of our protocols decreases when *h* decreases, where *r* < *h* ≤ *H*. Under the extreme case when *h = r +* 1, both our protocols achieve their minimum communication cost. Similarly, the security performance of both protocols decreases as *h* decreases. Since the number of random directed paths decreases as *h* decreases, the attacker can trace the source easily from the phantom location. However, *h* should be assigned with a reasonable value so as to balance the communication cost and the security performance.

Usually, *r*/*H* is no more than 1/5 for a large sensor network [9]. According to Section 6, if *r*/*H* ≤ 1/5, the communication cost of SLP and SLP-E are comparable, both of which grows as *h* grows. Table 2 shows that when 2 < *h* ≤ 20, the number of random directed paths increases significantly. In particular, the number of random directed paths increases drastically when *h* ≤ 20 but gradually slows down when *h >* 20. Table 2 shows that as *h* grows from 20 to 40, the number of random directed paths increases by only 6%. Thus, it is reasonable to define *h* = 20 to achieve a good balance between the communication cost and security performance.

## 7. Simulation Results

**Figure 4:**
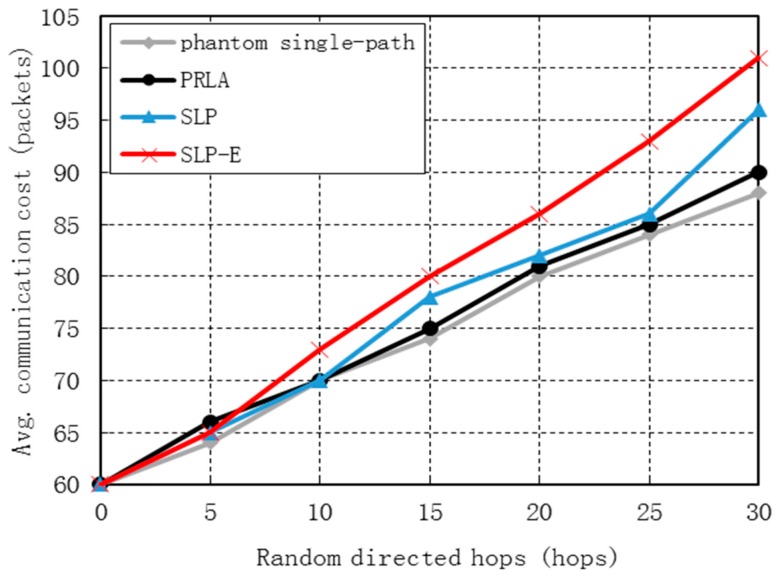
Communication cost *vs.* random directed hops.

**Figure 5:**
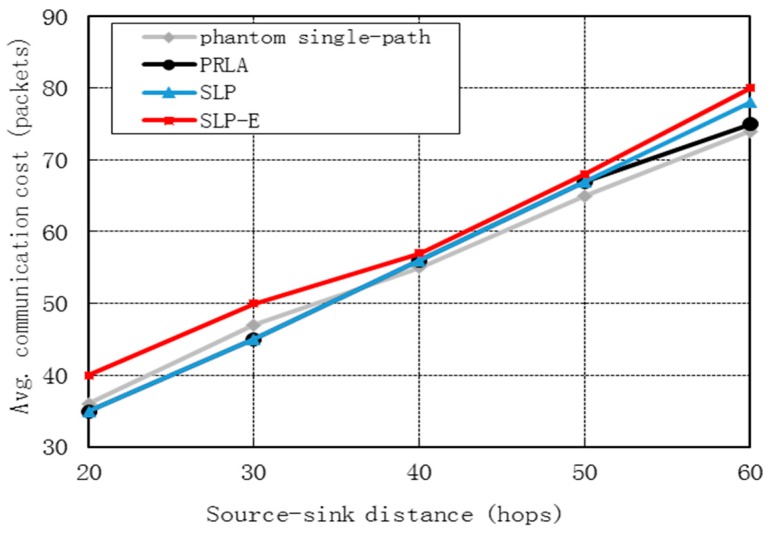
Communication cost *vs.* source-sink distance*.*

**Figure 6:**
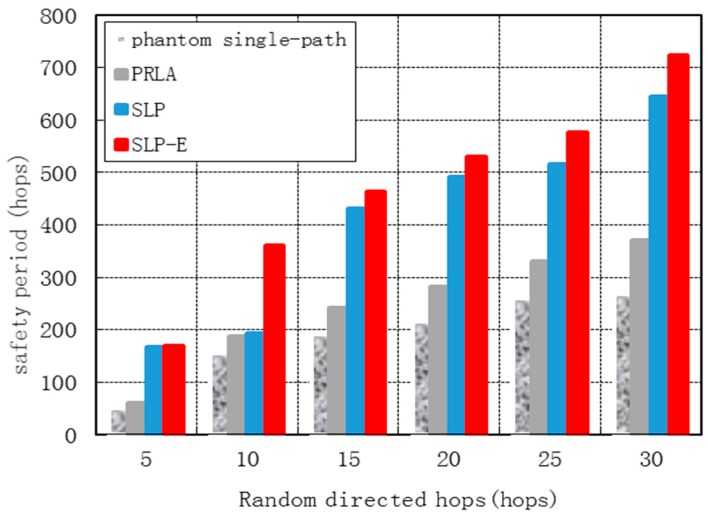
Safety period *vs.* random directed hops*.*

**Figure 7:**
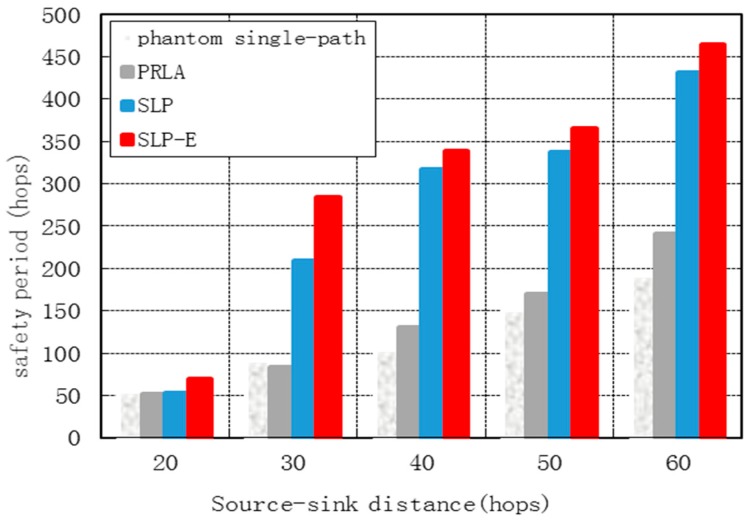
Safety period *vs.* source-sink distance*.*

We compare SLP and SLP-E with the typical source location protection protocols including phantom single-path [4] and PRLA [9] by OPNET. The network deployment is the same as [4,9]. Specifically, 10,000 sensors are distributed evenly over an area of 6000 × 6000 m^2^. In order to achieve random and even distribution of the sensors, we divide the monitored area into grids. Each sensor is located at the center of a grid at first. In order to generate a more reality sensor topology, we add a random and small perturbation *ε* to the location of each sensor, where *ε* is drawn from a normal distribution, *i.e.*, *ε ~* *N*(*μ*, *σ^2^*). The attacker always starts his tracing from the base station. The radius of the visible area of the attacker *r* is 6. The sensors that have no more than three neighbors take a percentage of 1% in all the deployed sensors. The base station is static and the source appears randomly. In Figure 4 and Figure 6, we set 60 to *H* and repeat the simulation for 50 times with different *h* to obtain the average result. Similarly, *h* is set to 15 in Figure 5 and Figure 7 and the simulation is repeated for 50 times with different *H* to obtain the average result. We adopt the safety period used in [4,7,9] to evaluate the security performance of the three protocols, which is defined as the number of hops before an attacker reaches the source [4,9].

### 7.1. Communication Cost

Figure 4 shows the communication costs for all four protocols grow as *h* grows. This is because the times of transmission for packets during the *h*-directed routing phase increases as *h* increases. The communication costs for phantom single-path, PRLA and our SLP are very close. The communication cost of SLP-E is higher than that of SLP. This is because bypassing the unsafe area during the shortest path routing process incurs extra transmissions. Specifically, compared with phantom single-path, which has the smallest communication cost, the communication for SLP-E increases by 8.26% on average and increases by 14.27% when *h* = 30. Figure 4 also shows that the communication cost for SLP-E increases merely by 3.91% on average when compared with SLP. Thus, the packet will be transmitted for four more times in SLP-E than SLP. Theory analysis in Section 6 indicates that *f_avg_* = 3 if *r*/*H* = 1/10. In conclusion, the simulation results is consistent with our theory analysis, which show that the increase amount of communication cost for SLP-E is acceptably low.

Figure 5 shows that the communication cost for all the four protocols grow as *H* grows and are very close. This is because as *H* grows, the distance from the source to the base station grows and consequently the number of transmission times needed for a packet to be transmitted from the source to the base station increases. Specifically, compared with phantom single-path and SLP, the communication cost for SLP-E merely increases by 6.50% and 4.98%, respectively.

### 7.2. Security Performance

Figure 6 shows the safety periods for all the four protocols grow as *h* grows. This is because as *h* increases, the phantom locations are farther from the source and consequently the number of random directed paths increases. The safety period for SLP-E increases by 147.68%, 91.58% and 15.58% on average when compared to phantom single-path, PRLA and SLP, respectively.

Figure 7 shows the safety periods for all protocols grow as *H* grows. This is because as the distance from the source to the base station grows, the attacker has to take more hops to reach the source. In particular, we observe the safety period for SLP-E increases by 145.44%, 114.94% and 7.71% on average when compared to phantom single-path, PRLA and SLP, respectively.

Figure 6 and Figure 7 show that SLP-E performs best and SLP outperforms PRLA in terms of safety period. The phantom single-path has the lowest security performance. The average safety period for SLP is significantly higher than with PRLA. In particular, the average safety period of SLP increases that of PRLA by nearly an order of magnitude. Figure 6 and Figure 7 also show that SLP-E provides a better security source location protection than SLP. This is because SLP-E can completely avoid inefficient path generation and therefore further improve source location security.

## 8. Conclusions

To monitor valuable objects sensor networks are usually deployed. To pursue profit from the objects, attackers generally capture the object by tracing the source. Therefore, many protocols have been proposed for source location protection. In this paper, we first analyze the limitations of existing works. Then, to address these limitations, we propose SLP to improve the source location security. Considering more powerful attackers with wider fields of vision, we further propose an enhanced protocol named SLP-E. Both theoretical analysis and simulation results show that compared with existing works, both SLP and SLP-E can improve the source location security significantly with low communication cost.
